# Internet searches offer insight into early-season pollen patterns in observation-free zones

**DOI:** 10.1038/s41598-020-68095-y

**Published:** 2020-07-09

**Authors:** Jane Hall, Fiona Lo, Shubhayu Saha, Ambarish Vaidyanathan, Jeremy Hess

**Affiliations:** 1 Department of Emergency Medicine, School of Medicine, University of Washington, 4730 University Way NE, Suite 104, #2021, Seattle, 98105 WA USA; 2 Department of Atmospheric Sciences, College of the Environment, University of Washington, 408 Atmospheric Sciences–Geophysics (ATG) Building, Box 351640, Seattle, WA 98195-1640 USA; 3 Rollins School of Public Health, Emory University, Grace Crum Rollins Building, 1518 Clifton road, Atlanta, GA 30322 USA; 4 School of Environmental Health, Emory University, 1518 Clifton road, Atlanta, GA 30322 USA; 5 School of Civil and Environmental Engineering, Georgia Institute of Technology, 790 Atlantic Drive, Atlanta, GA 30332-0355 USA; 6 Department of Emergency Medicine, School of Medicine, University of Washington, 4730 university way NE, Suite 104, #2021, Seattle, WA 98105 USA; 7 Department of Environmental and Occupational Health Sciences, School of Public Health, University of Washington, 1959 NE pacific St, Seattle, WA 98105 USA; 8 Department of Global Health, Schools of Medicine and Public Health, University of Washington, 4225 Roosevelt Way NE #100, Suite 2330, Box 354695, Seattle, WA 98105 USA

**Keywords:** Phenology, Ecological modelling

## Abstract

Tracking concentrations of regional airborne pollen is valuable for a variety of fields including plant and animal ecology as well as human health. However, current methods for directly measuring regional pollen concentrations are labor-intensive, requiring special equipment and manual counting by professionals leading to sparse data availability in select locations. Here, we use publicly available Google Trends data to evaluate whether searches for the term “pollen” can be used to approximate local observed early-season pollen concentrations as reported by the National Allergy Bureau across 25 U.S. regions from 2012–2017, in the context of site-specific characteristics. Our findings reveal that two major factors impact the ability of internet search data to approximate observed pollen: (1) volume/availability of internet search data, which is tied to local population size and media use; and (2) signal intensity of the seasonal peak in searches. Notably, in regions and years where internet search data was abundant, we found strong correlations between local search patterns and observed pollen, thus revealing a potential source of daily pollen data across the U.S. where observational pollen data are not reliably available.

## Introduction

Understanding regional airborne pollen patterns is important for a wide range of applications, including both basic and applied science in a range of domains from biology to ecology to public health. Estimates of pollen concentrations at a given location feed into agricultural, phenological, and ecological surveillance and models, and are incorporated into retrospective epidemiological analyses of associations between pollen exposure and adverse health outcomes like rhinitis and allergic asthma, which are of broad importance given that seasonal allergies affect 20–40% of the U.S. population^1, 2^. Observations are also important for driving forecast models that are used in risk communication to reduce pollen exposure in susceptible individuals.

Currently, the principle and most reliable source of pollen concentration information in the United States comes from the National Allergy Bureau (NAB), part of the American Academy of Allergy Asthma and Immunology (AAAAI). Pollen data included in the NAB dataset are collected by certified counting stations, where specially trained and certified allied health workers count pollen under the direction of an allergist^3^. Data must meet certain quality metrics to be included. While the data in this dataset are collected and reported using standardized methods, there is variability in sampling strategies, the costs of data collection are borne by the participating organizations, and participation varies over time. The totality of pollen count stations data currently listed on the AAAAI website covers less than 70 geographic locations in the continental U.S.^3^. Given these concerns, other valid approaches to generating estimates of airborne pollen that could expand spatial coverage and address some of the issues related to variable sampling time frames would be welcome for gaining insight into pollen season dynamics in regions without pollen monitors.

Among seasonal allergy sufferers who are sensitive to airborne pollen, a majority self-identify and self-treat allergy symptoms^4^. For this reason, we explored the potential for using Google Trends (GT), a web-based tool for quantifying popular interest in specific search terms, as a proxy for pollen observations in observation-free zones and for predicting pollen season dynamics. Our study builds on prior work examining associations between online search queries and real-life phenomena. Specifically, GT search data has been shown to correlate to outbreaks of West Nile Virus and respiratory syncytial virus, while the most well-known GT offshoot, Google Flu Trends, correlates strongly with official influenza surveillance data and frequently predicts major flu outbreaks^5–7^.

Relationships between GT searches and observed NAB pollen concentrations are a topic of great interest in the fields of allergy and ecology. Within the U.S., studies have examined this association on the scale of the U.S. as a whole, correlating also to allergic symptoms and antihistamine sales^8, 9^; as well as, state-wide in Texas from 2011–2012^10^. Outside of the U.S., authors have examined pollen and GT searches in Lisbon^11^, three regions in Germany^12^, and 21 regions in France, and have reported a wide range of associations from poor to excellent. Data from France showed that GT may be especially useful for identifying early spring tree pollen and grass pollen seasons, and poorer for identifying the later weed pollen season, though even associations during the early pollen season varied substantially^13^, suggesting site-specific factors that remain to be identified. Understanding these factors will be key to guiding appropriate GT data use and may be especially important in the U.S. due to the country’s large geographic area and heterogenous population.

Thus, the main goals of this study were to: (1) evaluate regional relationships between GT searches related to pollen and NAB pollen concentrations at diverse sites across the U.S., and (2) identify site-specific factors affecting association strength. In addition, we assess the potential for estimating the start of the pollen season using GT data. To these ends, we analyzed data from 40 location-matched GT Designated Market Areas (DMAs) and NAB stations both to determine the ability of GT data to accurately match NAB data, in the context of regional data availability, biogeography, and population. We restricted the scope of our investigation to early season pollen (January through June) when the largest peak in annual pollen concentration is commonly observed in the U.S. (mainly produced by trees); our analyses do not examine subsequent grass or weed pollen peak times which may have distinct characteristics^14^.

## Methods

### Data sources

NAB pollen concentration data were requested by mail from the AAAAI Executive Office. Total and taxa-specific pollen concentrations were received for 60 NAB certified stations, spanning 2003–2017, although only data collected in 2012 or later were used in this study. The list of participating sites and stations are listed in the acknowledgements. No imputation was done to fill in missing pollen concentration values.

GT data are freely available online and are reported as a random sample of historical Google search volume data. The data are adjusted by Google to fall between a range of 0–100 based on the highest value in each data sample accessed. Google does not quantify searches made by very few people (i.e., sets these values to “0”) and excludes duplicate searches (repeated searches by the same individual over a short period of time)^15^.

Biogeographical characteristics were gathered from multiple sources. Ecoregions were based on U.S. Environmental Protection Agency definitions^16^ and total annual precipitation and mean spring temperatures were calculated from daily values obtained from NASA’s Modern-Era Retrospective analysis for Research and Applications version 2 (MERRA-2) M2SDNXSLV, a satellite-based atmospheric reanalysis dataset^17^.

### Google trends data collection and preparation

Google Trends data on search volumes for the term “pollen” from 2012–2017 with daily resolution were accessed using *pytrends*, an open-source, user-created version of an Application Programming Interface that allows programs created in the language Python to directly communicate download parameters with GT servers https://github.com/drjanehall/GTDailySearches^18^. Other GT search terms previously described in the literature related to pollen, including “pollen count” and “pollen allergy” were examined visually via the GT web app, but were not ultimately used in analysis due to low overall search volumes and lack of seasonal peaks (see Supplementary Fig. 1A,B for representative examples and Supplementary Table 1 for the full list of candidate search terms). Geographical parameters for data downloads were specified for each Nielsen Designated Market Area (the smallest search region available for GT data) that most closely matched available NAB pollen count data (for a maximum of 0.3 latitude or longitude decimal degrees of distance to be considered a match). All available NAB stations data with associated DMA matches were examined with the exception of Twin Falls, ID which had exceptionally sparse GT data representing less than 10 daily data points per year. See Supplementary Table 2 for the complete list of regions used in the analyses. All downloads were performed with 10 × replicates and averaged to compensate for inter-download variation (See Supplementary Fig. 2A,B for visualization of variation between downloads).

### Statistical analyses

#### Inclusion criteria

NAB data were evaluated on the level of station-year. Since stations are directed by the NAB to collect and measure pollen at least three times per week, we excluded stations based on two metrics serving as proxies for compliance: percent days missing collections and longest gap in between collections during the pollen season. Station-years missing pollen concentration records for more than 60% of days per year were excluded from analyses. Station-years with over four consecutive days missing pollen concentration records within 10 days before or after the first “high” pollen concentration day were also excluded from analyses. Pollen concentrations of at least 200 grains/m^3^ were considered “high”, as frequently defined^19^.

#### Assessment of data quality

Data quality was assessed for NAB and GT data with a focus on missing data (total and consecutive days of missing data). Regional factors were also screened for association with data quality via Pearson’s correlations; these included ecoregion classification, total annual precipitation, mean spring temperatures, latitude, longitude, and media consumption (TV-homes per DMA region). Factors identified as associated with data quality were then evaluated using scatter plot visualization and univariate linear regression to assess magnitude and direction of the associations. Comparisons between groups were done via Student’s t-tests.

#### Relationship between GT and NAB data

GT and NAB data for the first half of the calendar year (January to June) were visually compared using line plots for search volumes vs. pollen concentrations over time. NAB data were normalized to a maximum value of 100 per year for comparison to GT data. Further, GT and NAB data were log-transformed and lightly smoothed using locally weighted scatterplot smoothing (LOWESS) methods, via the *lowess* package in Stata with a bandwidth of 0.1. Bandwidth is a smoothing parameter ranging from 0–1, where lower values correspond to less smoothing (See Supplementary Fig. 3A,B for a representative plot before and after smoothing). Log-transformed and lightly smoothed data were then compared using Spearman rank correlation to generate a rho value quantifying the strength of the ordinal relationship between NAB and GT data. Correlations were also examined in the context of (1) percent days per year that GT-adjusted search volumes were equal to zero, and (2) seasonal peak signal-to-noise ratio. Peak signal-to-noise ratio was approximated by comparing Lowess smoothed GT data (bandwidth = 0.8) and untransformed GT data, and defined as the mean average difference. Similar strategies for peak detection have been previously used in such settings as fire history detection in sediment charcoal records for ecological study and detection of pulsatile secretions of luteneizing hormone in endocrinology^20, 21^.

#### Pollen season start dates

In the absence of academic consensus or regulatory guidelines to define pollen season start dates, current definitions used in the literature vary widely^22^. Most commonly, the start of the pollen season is the date upon which a predefined threshold is met, based on either: (1) a percentage of annual pollen, (2) a certain daily pollen concentration (or over a predefined period, such as three days), or (3) a number of consecutive days during which pollen grains are recorded^23, 24^.

In order to address this issue, we tested multiple definitions from recent literature and assessed their concordance when applied to NAB data. These were: the date that (1) cumulative pollen count reached 5% of annual total^25–27^, (2) cumulative pollen reached 2.5% of annual total^28, 29^, and (3) four consecutive days of pollen grains were recorded^30^. In addition, we examined the date that total daily pollen concentration exceeded 200 grains/m^3^, which is sometimes considered a threshold relevant to clinical symptoms, and thus potentially also relevant to the internet search activity of allergy sufferers^31^. However, defining symptom thresholds is itself a challenge, since both the ways in which the presence and severity of symptoms manifest, and are recorded, can change across individual experiences and study definitions^32, 33^.

In line with previous literature, we found major differences in season start date between definitions—both the magnitude and directionality of the difference were heterogenous by region and year^24^. However, of the definitions tested, we eventually selected cumulative pollen reaching 5% of annual total as the criteria for this study, due to the fact that it was found to overlap most closely with the first date of absolute pollen concentrations reaching 200 grains/m^3^ and thus potentially more likely to be reflected in Google searches (Supplementary Fig. 4A–C).

Pollen season start dates calculated from NAB data and those calculated from GT data were compared using univariate analysis.

### Software

Stata IC version 15.1 (College Station, TX, USA) was used for all data analyses. DMA characteristics and GT data were downloaded using Python version 2.7.10 (Python Software Foundation). All Stata code used for analysis and Python scripts used for GT downloads are available upon request.

## Results

### Assessment of data quality

#### National Allergy Bureau pollen concentration data quality

To assess the quality of NAB data overall, we analyzed gaps in data recording and percentages of missing data in daily NAB measurements from each station from January to December of each year. Availability of pollen concentration data varied widely by station, with percent of days per year missing pollen data ranging from 0% (e.g. San Antonio, TX; 2012) up to 100% (e.g. Oklahoma City, OK; 2014) (Supplementary Fig. 5A). Common days missing data were at the beginning of the year, the end of the year, and on weekends (data not shown). Although NAB directs its certified pollen counting stations to collect data for a minimum of 3 days per week, gaps in pollen collection within 10 days before and after the first recorded high pollen concentration (200 grains/m^3^) spanned up to 10 consecutive days (Supplementary Fig. 5B). Over the span of the year, the median gap between measurements across station-years was 5 days (IQR = 3.12). The date of first available pollen concentration data ranged from day 1 of the year to day 96 with a median day of 3 (IQR = 1.27) (Supplementary Fig. 5C). For the majority of station-years (64.5%), the first day of the first recorded data for the year was the same as the first day with a non-zero pollen count.

#### Google trends search data quality

We analyzed GT daily data quality per DMA region during the early pollen season, from January to June of each year. The percent of missing days of GT data ranged from 0–93% (lowest missing from San Jose CA 2013 and highest missing from Midland TX 2012, respectively) with median and IQR = 33% (8–51%) (Supplementary Fig. 6A). Earlier years of GT data had more daily search volumes not quantified (referred to here as “missing”) due to lower search volumes and not meeting Google’s threshold for inclusion (Supplementary Fig. 6B). Variation was observed between GT download iterations, as GT provides a random sample of its data for each download (Supplementary Fig. 2A,B).

### Factors associated with data quality

Biogeography and population characteristics were assessed for their impact on data quality, specifically overall ecoregion classification, total annual precipitation and mean spring temperature (chosen for their likely impact pollen production and seasonality^34^), as well as TV-homes, a combinatorial metric for population size and media use.

With respect to ecoregion, the majority of NAB stations were classified as Eastern Temperature Forests (67.6%) or Great Plains (21.6%). Other ecoregions each represented 5% or less of NAB stations: Marine West Coast Forest, Mediterranean California, and Northwestern Forested Mountains. U.S. ecoregions not represented by NAB stations included: Northern Forests (as in Vermont), Tropical Wet Forests (as in southern Florida), North American Deserts (as in Nevada), Southern Semi-Arid Highlands (as in southeastern Arizona), and Temperate Sierras (as in southwestern New Mexico). As a whole, NAB stations in Great Plains ecoregions had slightly higher data quality (p < 0.01), with a median of 70.7% days of non-missing data (IQR = 60.3%, 89.9%), versus Eastern Temperate Forests with a median of 63.8% (IQR = 53.2%, 68.4%) across station-years. Statistical comparisons were not performed between other ecoregions due to small sample sizes, however data by ecoregion can be viewed in Supplementary Fig. 7.

With respect to climactic factors, we evaluated mean spring temperatures, total annual days of precipitation, latitude, and longitude, in relation to percent of missing GT and NAB data as well as number of consecutive days of missing NAB data. Among all pairwise comparisons, a few significant relationships were identified. Mean spring temperature (°C) exhibited a positive correlation with non-missing NAB data (% days) [R-squared = 0.29, Coefficient = 1.89 (95% CI 1.31, 2.46), Supplementary Fig. 8]. This may reflect the behavior described by some pollen counting stations in northerly, colder regions of not recording or monitoring pollen until weather is warmer and pollen is more likely to be produced (from personal correspondence, data not shown). Mean spring temperature was strongly inversely correlated with latitude, as is expected (R^2^ = 0.73). Total annual days of precipitation was found to be positively correlated with longitude (R^2^ = 0.38; Coefficient = 1.39; 95% CI 1.04, 1.73), which is consistent with Köppen–Geiger dry-moist climate classifications for the continental U.S.^35^. No associations were detected between any other climactic factors or data quality metrics examined via univariate regression analyses (R^2^ ≤ 0.1).

With respect to regional population sizes and media consumption, the percent of non-missing NAB pollen concentration data was not found to be correlated (as estimated by number of TV-homes in the associated DMA region; p = 0.29; R^2^ < 0.01). In contrast, the percent days of missing GT data was strongly correlated to the log-transformed number of TV-homes in the region [Coef = − 22.6 (95% CI − 24.7, − 20.6); p < 0.01; R^2^ = 0.68] (Supplementary Fig. 9).

### Correlation between NAB and GT data with respect to data quality

#### Data quality inclusion criteria for correlation analyses

NAB total pollen concentrations from the majority of station-years showed a bimodal seasonality consisting of one larger peak early in the year and one smaller peak later in the year (See Supplementary Fig. 1 for national seasonality). For correlation analyses, we focused specifically on the period from January to June to examine the extent to which GT data correlated to the larger, early season peak in total pollen. Of 246 GT location-matched NAB station-years, 24 (9.7%) had no NAB data recorded in the period of interest. In addition, the following station-years did not meet data quality inclusion criteria: 85 (34.5%) station-years had over 60% of days missing data during the pollen season, and an additional 32 (13.0%) station-years had over four consecutive days missing data within 10 days of the first high pollen concentration day of the year. A total of 105 station-years, representing 27 NAB stations, were ultimately included in correlation analyses. See the Supplement for visualizations of ecoregions (Supplementary Fig. 10) and geographical distribution (Supplementary Fig. 11; Interactive Map https://bit.ly/2XTlHrC)^36^ of NAB stations represented in the included study sample.

#### Effects of data missingness on NAB-GT correlation strength

**Figure 1:**
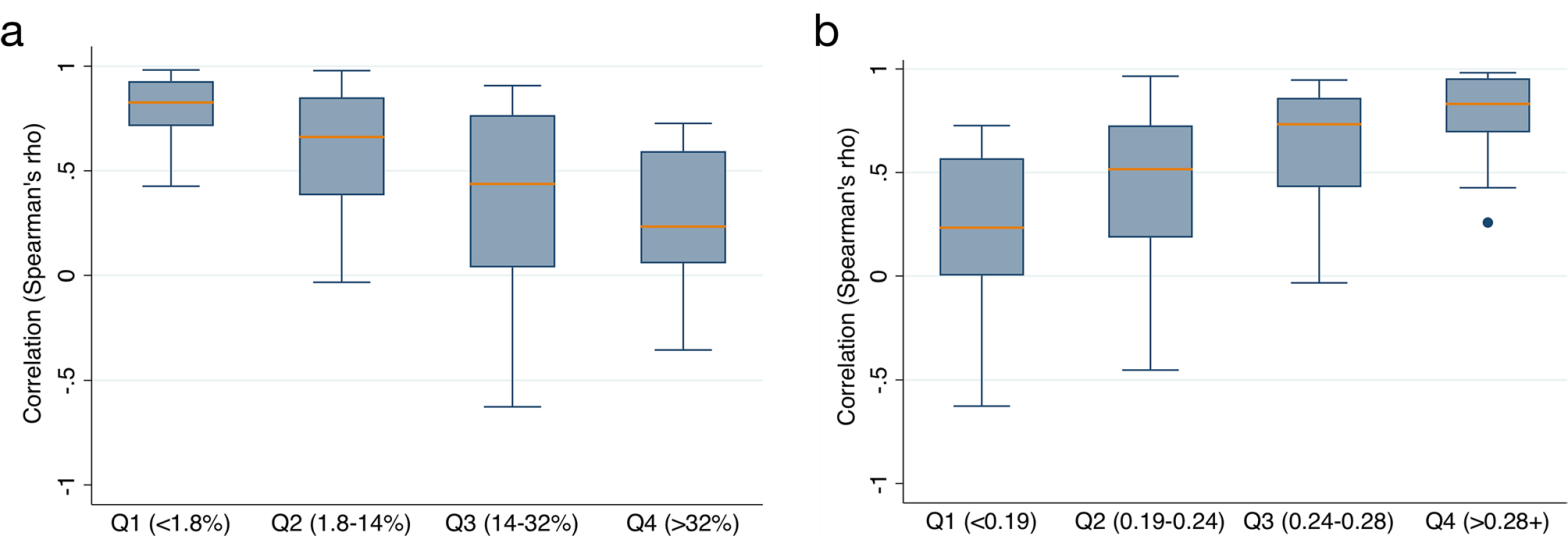
Correlation between Google Trends searches and National Allergy Bureau pollen concentration data with respect to data quality and pattern. (**a**) Correlation by quartiles of annual percent of missing Google Trends data. (**b**) Signal to noise ratio (size of peak relative to smoothing function).

Daily total pollen concentrations from NAB data were compared to daily GT search counts by station-year via Spearman rank correlation. Since GT data varied widely with respect to percent of missing days of data per year, station-years were grouped into quartiles to test the effect of missingness on ability of GT to correlate with NAB data, with quartile cutoffs at 1.8%, 14%, and 32% of days missing GT data. Significant differences were identified in correlation strength between Q1 v. Q2 (p < 0.01) and Q2 v. Q3 (p = 0.02). Rho values by quartile were: Q1 0.71 (IQR 0.83, 0.93), Q2 0.66 (IQR 0.38–0.85), Q3 0.44 (IQR 0.04–0.77), Q4 0.23 (IQR = 0.06, 0.59) (Fig. 1A).

#### Effects of GT peak signal strength on GT-NAB correlation strength

As a proxy for site-specific estimates of signal to noise ratio and ability to identify peaks in GT data, the mean absolute difference between daily GT adjusted search volumes (“signal”) and a heavily smoothing lowess function (baseline fluctuations or “noise”) was calculated per station year (Supplementary Fig. 12A). Station-years were separated into quartiles to test the effect of signal strength on ability of GT to correlate with NAB data, with cutoffs at 0.19, 0.24, and 0.28. Significant differences were identified in correlation strength between Q2–Q3 (p = 0.04) and Q3–Q4 (p = 0.02). Spearman’s rho values by quartile were: Q1 0.23 (IQR 0.00, 0.57), Q2 0.52 (IQR 0.19–0.73), Q3 0.73 (IQR 0.43–0.86), Q4 0.83 (IQR 0.69, 0.95) (Fig. 1B). Correlation between GT peak signal strength and GT-NAB correlation strength can also be visualized by scatter plot (Supplementary Fig. 12B).

### Effects of ecoregion and climate on GT-NAB correlation strength

When comparing the two main ecoregions represented in the study sample, correlations appeared to be somewhat weaker (p = 0.01) in Great Plains locations, among which the median rho value was 0.70 (IQR 0.39, 0.90) than in Eastern Temperate Forest locations, among which the median rho was 0.44 (IQR 0.13, 0.73). Other ecoregions were not compared due to small sample sizes, but correlation data by ecoregion are reported in Supplementary Fig. 7. We were not able to detect statistically significant associations between either precipitation or spring temperatures with GT-NAB correlation strength, although numbers were small (e.g., N = 18 stations in 2013). However, scatter plot visualization indicate that GT-NAB correlation strength may tend toward positive associations with annual precipitation and negative associations with spring temperature (Supplementary Fig. 13A,B).

#### Variation in correlation strength across sites

**Figure 2:**
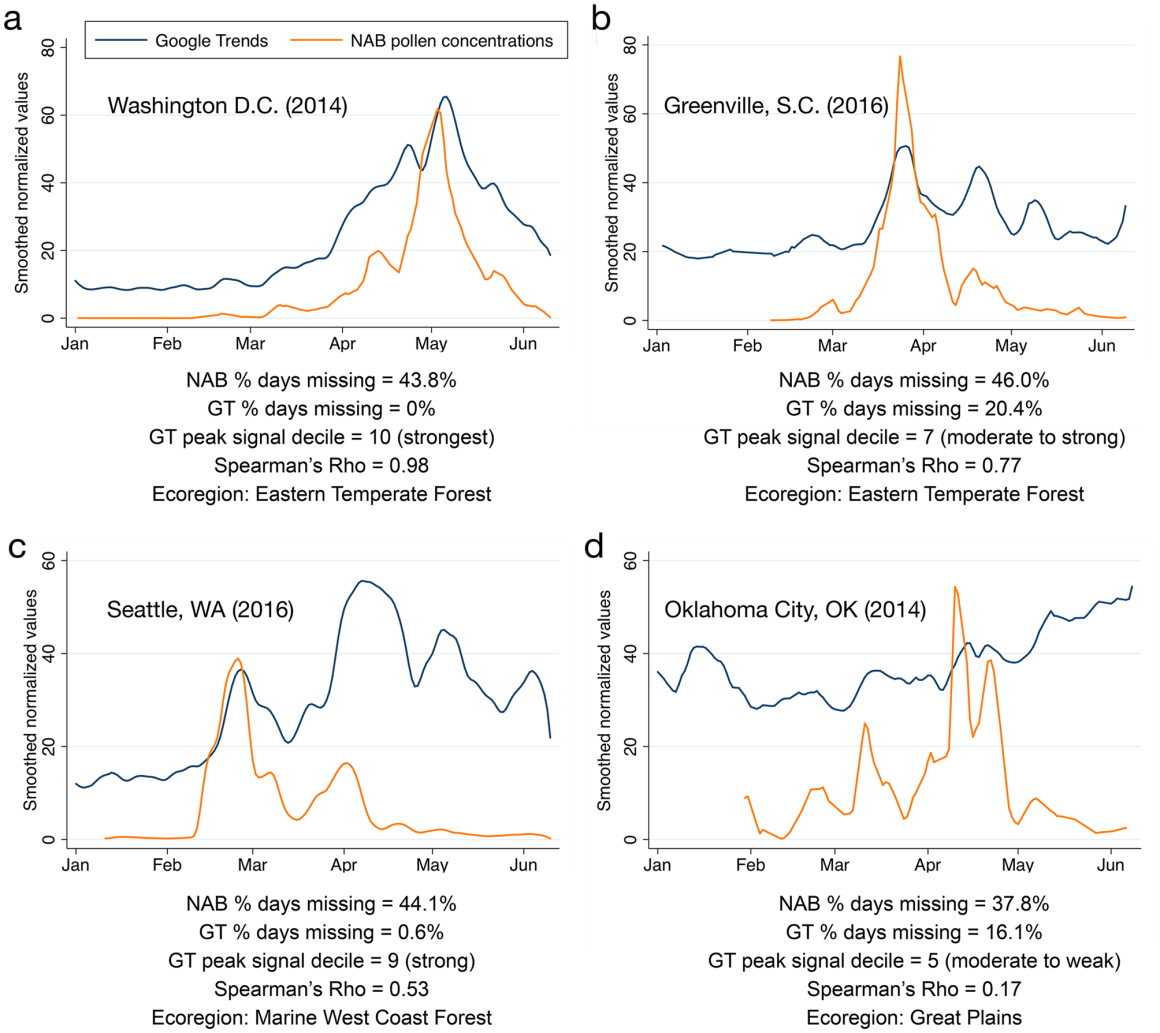
Overlay of lightly smoothed, normalized Google Trends search data (blue) and NAB pollen concentration data (orange) for representative station-years. Examples of (**a**) excellent, (**b**,**c**) good to moderate, and (**d**) poor correlation between GT and NAB data.

Overall, comparisons between GT search data with any amount of non-missing data and NAB pollen concentration data resulted in Spearman’s rho values that ranged from very poor (rho = − 0.63) to excellent (rho = 0.98) for 105 station-years, covering 27 unique stations (see Fig. 2A–D), with a median rho of 0.24 (IQR 0.61–0.80). See Supplementary Table 2 for complete data missingness and rank-correlation values by station-year.

### Season start estimates

To evaluate whether Google Trends data on search volumes for “pollen” could be used to estimate the start of the pollen season, we compared GT-calculated to NAB-calculated season starts, where start date was defined as the first date that pollen concentrations reached 5% of total annual cumulative pollen (see “Methods” for rationale and additional context).

#### Effects of data transformations and data quality thresholding on estimation accuracy

**Figure 3:**
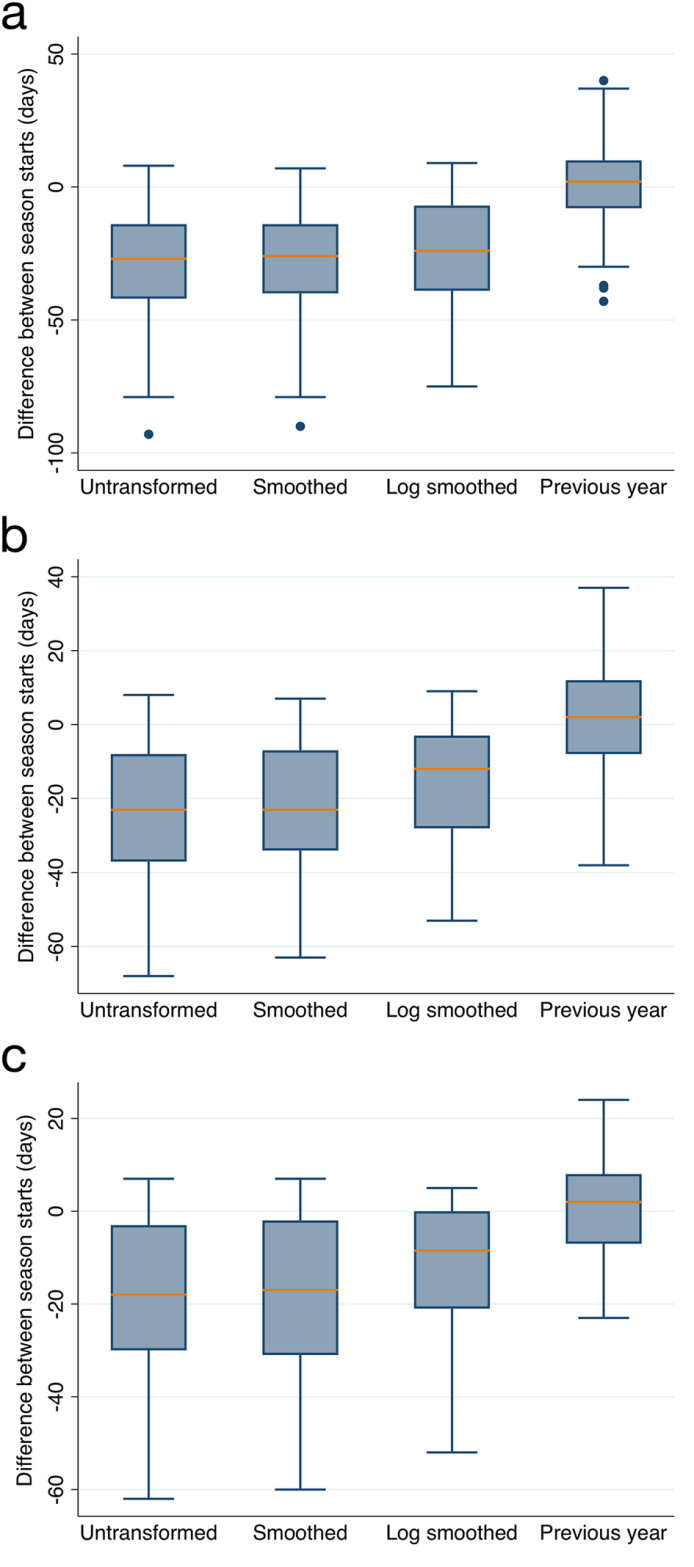
Difference in days between Google Trends- and NAB-calculated season start dates. Differences in start dates are shown for untransformed, smoothed, and log-transformed smoothed data. As a reference, differences between NAB-calculated start dates those calculated from NAB data for the previous year dates are displayed as well, for (**a**) All available station-years, (**b**) additional inclusion criteria of NAB data collection beginning within first month of the year applied, (**c**) additional inclusion criteria of < 20% missing GT data applied.

Estimates derived from GT data were examined in the context of percent days missing and first date of available NAB data (Fig. 3A–C and Supplementary Table 3). Overall, comparing smoothed GT and NAB data, and comparing log-transformed smoothed GT and NAB data decreased the discrepancies between GT-derived and NAB-derived estimates of season start dates, as compared to using untransformed data. As a result of applying progressive inclusion criteria based on data quality, the range of discrepancies between NAB- and GT-derived data also decreased. When examining all station-years using smoothed, log-transformed data, GT-derived start estimates preceded NAB-derived start dates by a median value of − 24 days (IQR − 39, 7). With progressive inclusion criteria applied, this decreased to − 12 days (IQR − 28, − 3) and then to − 8.5 days (IQR − 21, 0). NAB-derived start dates using data from the previous year had discrepancies from the current year with a median of 2 days, and in IQR within 1–2 weeks.

## Discussion

NAB station locations are limited due to the requirement of needing specially trained and certified allied health workers who must dedicate 2 hours a day, three times a week to counting pollen, as well as an allergist to oversee pollen counting. GT data can be a useful source of publicly available user-derived data related to pollen allergy patterns in regions where NAB are not available, though there are limitations on the extent to which GT can serve as a reliable proxy measure.

**Figure 4:**
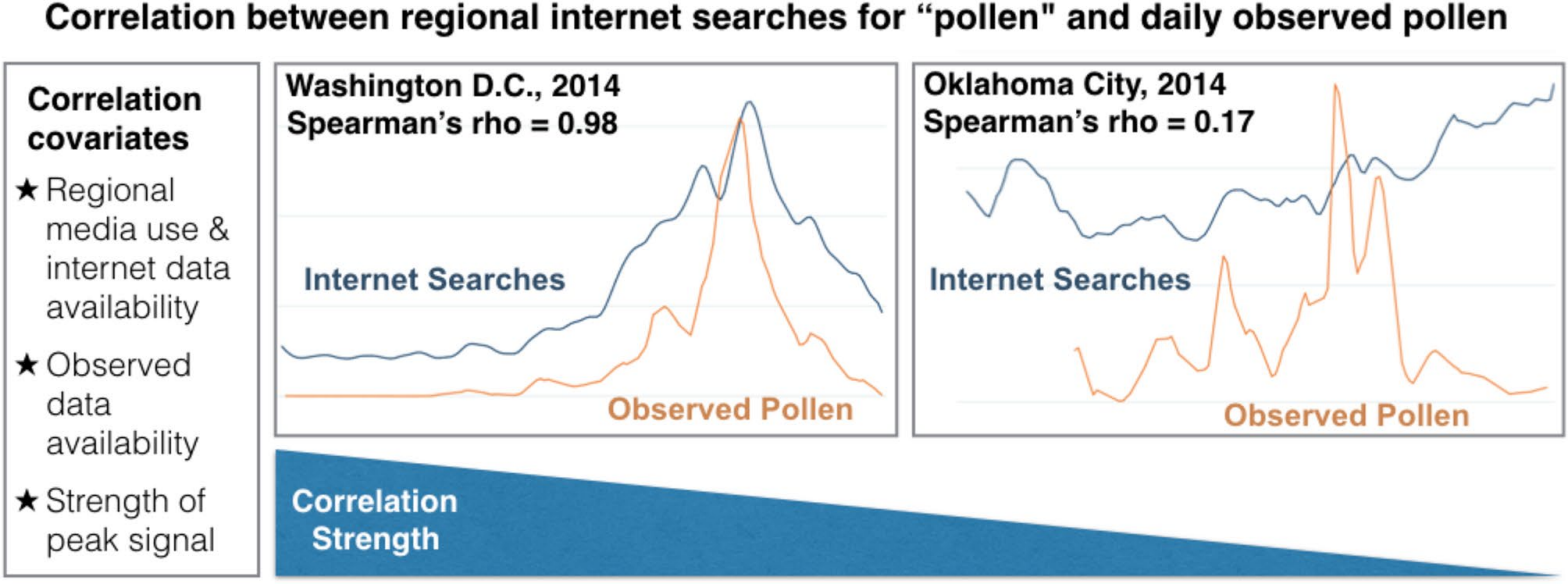
Summary of findings: covariates related to strength of correlation between regional internet searches and observed pollen, with representative examples. Line graphs show lightly smoothed normalized values for both Google Trends search volumes and daily observed pollen concentrations.

We found that associations between NAB early season total pollen concentrations and GT search volumes for “pollen” are moderate to excellent in many regions, with one important factor being the percent of missing GT data. We find that DMA rank (a proprietary metric from The Nielsen Company to approximate large population size and media consumption in terms of TV-homes) is directly related to percent of missing data (see Supplementary Fig. 14). This suggests that regions with more people performing Google searches give rise to GT reports with less missing data, which is logical given that Google uses a threshold to set low volumes of searches to zero. A second important factor affecting associations is peak signal strength in GT data. Peak signal strength may be both an indicator of signal to noise ratio intrinsic to GT data, and a marker of regions with a distinct and large early season pollen peak that can be more well captured by Google searches; regions with lower peak signal strength had poorer associations with NAB data (Fig. 4). Indeed, percent missing data and signal strength in GT data are strongly correlated for many regions (Supplementary Fig. 12B).

Of the top 50 Nielsen ranked DMAs, which are likely have adequate population size and media consumption for approximating NAB data, at least 25 could provide GT data in NAB observation-free zones (Supplementary Fig. 11; Interactive map may be accessed at https://bit.ly/2XTlHrC)^36^. In addition, the utility of GT data as a proxy for observed pollen pattern information should improve over time as more search use leads to more search volume and less data exclusion, following our observed trends of improved data quality over time.

In addition to re-assessing the relationships outlined here as online search volumes increase over time, there are other potential applications based on our findings. For example, although currently using GT data to identify pollen season start dates tends to precede the NAB-derived start dates, the precision of GT-derived start dates appears to comparable to start dates calculated from previous year data. Thus, GT-derived data may be useful for approximating true season starts either with lead-time in mind or in more advanced modeling that includes factors affecting lead time such as climate-based data. In this way, GT data may be helpful in the future for estimating a large number of historical and current location-specific data points for annual pollen season start dates over time, and this data could be used to investigate varied trends alongside other data, including weather, plant phenology, health, or other GT data to assess co-variance between variations in pollen season start dates and other trends like the effects of climate change over time (comparing to local temperatures or extended spring indices, for example).

One caveat of our findings is that the underlying reason for GT search results demonstrating an association with NAB pollen concentrations are unknown. That is, Google users may search for pollen-related terms due to concerns or questions related allergic symptoms, or for unrelated reasons such as observed pollen release; searches may be at first onset or with continuing interest or symptom persistence, and with or without a dose-dependent relationship. In the setting of allergy sufferers, particularly those who are aware of their diagnosis and the etiology of their symptoms, the threshold for performing Google searches related to pollen are unknown, and much of the population likely experiences seasonal allergies with varying sensitivity to pollen doses and in response to varying allergenic plant taxa. Factors that can affect the relationship between pollen and symptoms include the sensitization history of the individual, concomitant asthma or respiratory infections, climactic factors such as humidity, and exposure to risk factors such as agricultural pesticides^37–39^.

As noted, our analyses do not extend to pollen produced in the second half the year, including pollen produced by many grasses and weeds, which can affect allergy sufferers differently than the tree pollen that is commonly produced in the spring. An additional consideration is that airborne pollen concentrations may not directly relate to allergen exposure, as pollen potency can change dynamically throughout the season^40^. It should also be noted that air pollution can also cause rhinitis and other pollinosis-like symptoms that could in turn drive internet search activity wholly independent of pollen concentrations. Therefore, researchers that plan to use GT searches to estimate pollen concentrations should account for air quality measures such as ozone and particulate matter concentrations^41^. Finally, with respect to search data, Google reserves the rights to change its search algorithms at any time and without notice, which could change the relationship between pollen-related searches and actual pollen concentrations and limit reproducibility of the findings presented here.

## Conclusion

GT data may be helpful for examining annual pollen patterns and estimating the start of the pollen season in regions that currently lack data on actual pollen concentrations. There is potential for using GT data to extend or in lieu of pollen data observations, particularly when limitations in the relationship between GT data and pollen observations are taken into account.

## Supplementary information

Supplementary information

## Data Availability

Data from the National Allergy Bureau is available upon written request and may be released directly by the pollen counting station or by the AAAI Executive Office. Visit https://www.aaaai.org for current data release guidelines. Data downloaded by the authors from Google Trends for this study can be accessed in its original form at Mendeley Data (https://dx.doi.org/10.17632/xpy7jykfzw.1). The Python script used for downloading Google Trends data can be accessed at https://github.com/drjanehall/GTDailySearches. *Data sources* National Allergy Bureau data for the analyses in the study were provided by the following stations (the associated professionals and clinics for each station are listed below). Stanley M Fineman, MD MBA FAAAAI, Atlanta Allergy and Asthma Clinic, Marietta (Atlanta), GA. Sheila Amar, MD, FAAAAI, FACAAI, Allergy & Asthma Center of Georgetown, Austin, TX. Jonathon Matz, MD, FAAAAI, & David Golden, MD, FAAAAI, Baltimore, MD. Linda Ford, MD, FAAAAI, The Asthma and Allergy Center, PC, Bellevue, NE. David Weldon, MD, FAAAAI, FACAAI, Scott & White Clinic, College Station, TX. Robert Nathan, MD, FAAAAI, & Daniel Soteres, MD, MPH, FAAAAI, Asthma and Allergy Associates, PC, Colorado Springs, CO. Donald Pulver, MD, FAAAAI, Allergy, Asthma & Immunology of Rochester, Rochester, NY. Andy Roth, RAPCA, Dayton, OH. Duane Harris, MD, FAAAAI, Intermountain Allergy & Asthma Clinic, Draper, UT. Philip Gallagher, MD, FAAAAI, Allergy & Asthma Associates of Northeastern Pennsylvania, Erie, PA. Kraig Jacobson, MD, FAAAAI, Allergy & Asthma Research Group, Eugene, OR. Neil Kao, MD, FAAAAI, Allergic Disease and Asthma Center, Greenville, SC. Tony Huynh, City of Houston, Houston, TX. Jay Portnoy, MD, FAAAAI, Children’s Mercy Hospital, Kansas City, MO. James Anderson, MLT, OSHTECH, London, ON. Robert Bush, MD, FAAAAI, University of Wisconsin Medical School, Madison, WI. Joseph Leija, MD, FAAAAI, Melrose Park, IL. Harold Kaiser, MD, FAAAAI, Clinical Research Institute, Minneapolis, MN. Warren Filley, MD, FAAAAI, OK Allergy Asthma Clinic, Inc., Oklahoma City, OK. Martha Tarpay, MD, Allergy & Asthma Center, Oklahoma City, OK. Wayne Wilhelm, Saint Louis County Health Department, St. Louis, MO. Robert Gomez, Wiford Hall Ambulatory Surgical Center, San Antonio, TX. Alan Goldsobel, MD, FAAAAI, & James Wolfe, MD, FAAAAI, Allergy and Asthma Associates of Northern California, San Jose, CA. Frank Virant, MD, FAAAAI, Northwest Asthma & Allergy Center, Seattle, WA. Rhizza Adams, Springfield-Greene County Health Department, Springfield, MO. James Love, Jr., MD, PhD, FAAAAI, Allergy Clinic of Tulsa, Tulsa, OK. Richard Henry, MD, Asthma & Allergy of Idaho, Twin Falls, ID. Pramila K. Daftary, MD, FAAAAI, Allergy & Asthma Care of Waco, Waco, TX. Susan E. Kosisky, MHA, US Army Garrison-Forest Glen, Silver Spring, MD (Washington D.C.). Christopher Randolf, MD, FAAAAI, Waterbury, CT. Michael Nickels, MD, PhD, Allergy and Asthma Consultants, Inc., York, PA.
