# Supplementary material for: Internet searches offer insight into early-season pollen patterns in observation-free zones
Source: Sci Rep. 2020 Jul 9;10:11334. doi: 10.1038/s41598-020-68095-y (PMC7347639; doi:10.1038/s41598-020-68095-y)
Supplement: Supplementary file 1 — Supplementary information [file 41598_2020_68095_MOESM1_ESM.pdf]

Supplementary Information for "Internet Searches Offer Insight into Early-Season Pollen Patterns in Observation-Free Zones"

Title

Internet Searches Offer Insight into Early-Season Pollen Patterns in Observation-Free Zones

Authors

Jane Hall PhD* [a], Fiona Lo MS [b], Shubhayu Saha PhD [c], Ambarish Vaidyanathan PhD [d,e], Jeremy Hess MD MPH [f,g,h]

*Corresponding author.

[a] Affiliation: Department of Emergency Medicine, School of Medicine, University of Washington, Mailing address: 4730 University Way NE, Suite 104, #2021, Seattle, WA 98105. Phone: +1 (203) 441-3281. Fax: +1 (206) 299-0759. Email: janehall@uw.edu.

[b] Affiliation: Department of Atmospheric Sciences, College of the Environment, University of Washington. Mailing address: 408 Atmospheric Sciences–Geophysics (ATG) Building, Box 351640, Seattle, Washington 98195-1640. Email: fionalo@uw.edu.

[c] Affiliation: Rollins School of Public Health, Emory University. Mailing address: Grace Crum Rollins Building, Atlanta, GA 30322. Email: shubhayu.saha@emory.edu.

[d] Affiliation: School of Environmental Health, Emory University.

[e] Affiliation: School of Civil and Environmental Engineering, Georgia Institute of Technology, 790 Atlantic Drive, Atlanta, GA 30332-0355. Email: av41@ce.gatech.edu.

[f] Affiliation: Department of Emergency Medicine, School of Medicine, University of Washington.

[g] Affiliation: Department of Environmental and Occupational Health Sciences, School of Public Health, University of Washington.

[h] Affiliation: Department of Global Health, Schools of Medicine and Public Health, University of Washington. Mailing address: 4225 Roosevelt Way NE #100, Suite 2330, Box 354695, Seattle, WA 98105. Email: jjhess@uw.edu.

Supplementary Figures

Supplementary Figure 1A. Seasonality of Google searches for some candidate terms compared to NAB pollen concentration data (orange), across the United States in 2016.

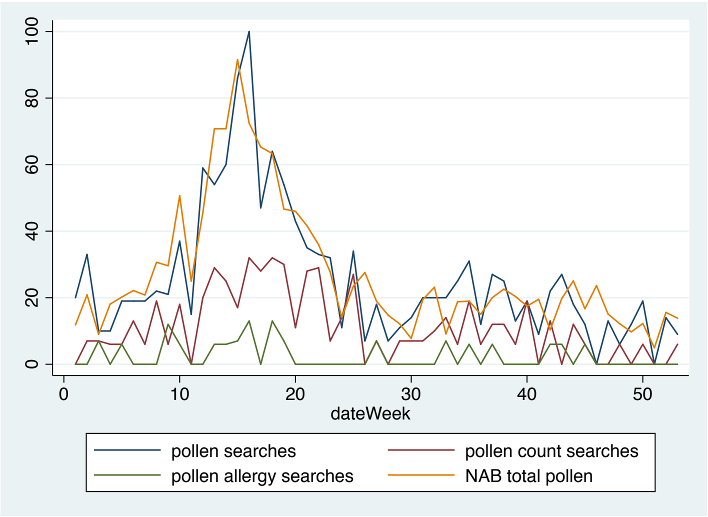

Supplementary Figure 1B. Low search volumes for some candidate terms, relative to “pollen”, across the United States in 2016. Pollen (yellow), rhinitis (blue), runny nose (red), ocular allergy (green), pollen allergy (purple)

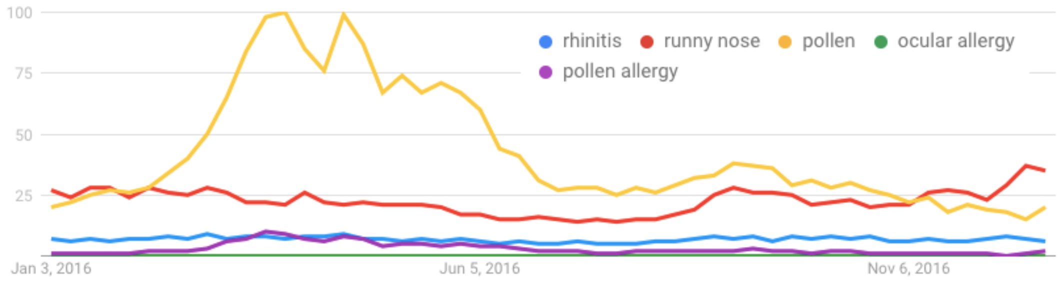

Supplementary Figure 2. GT download variation for representative station-year (Atlanta, GA 2015)

A. Overlay of three individual downloads B. Mean and 95% CI for 10 downloads.

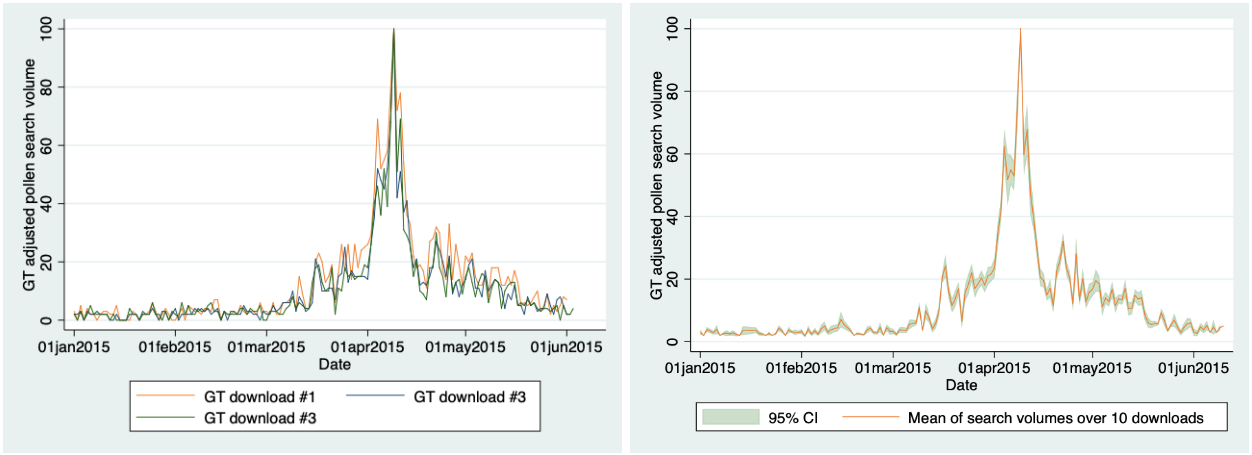

Supplementary Figure 3. Lowess smoothing for representative station-year (Atlanta, GA 2014)

A. Before smoothing B. After smoothing

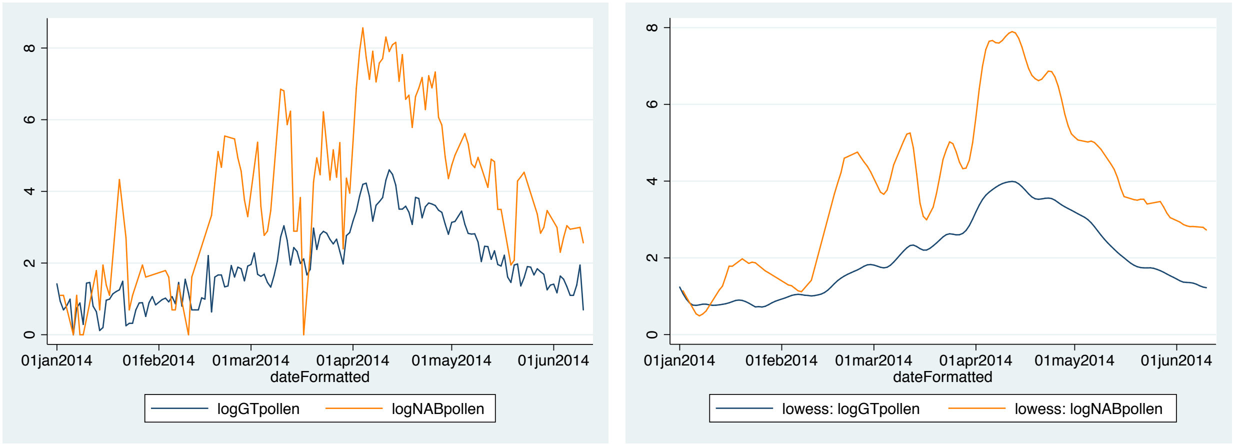

Supplementary Figure 4. Percent discrepancies between season start date definitions (baseline definition is first date where absolute pollen concentration reaches 200 grains/m3).

A. Four consecutive days of non-zero data

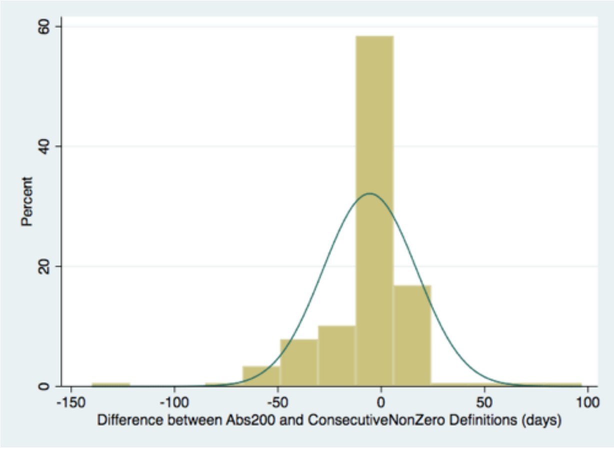

B. Cumulative pollen reaches 5% of annual sum

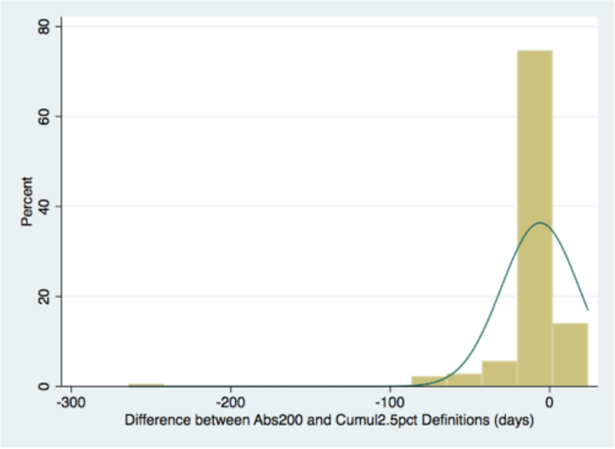

C. Cumulative pollen reaches 2.5% of annual sum

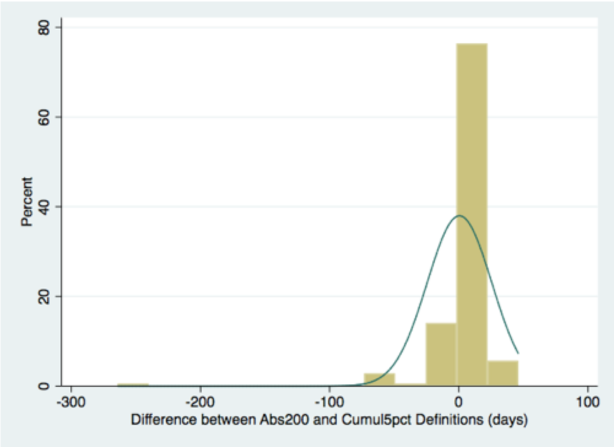

Supplementary Figure 5. NAB data quality (frequency counts are of station-years): A. Percent missing days over the year, B. Gaps (consecutive days) in NAB data collection, C. Days before first available data, from January 1.

A. Percent Missing NAB Data

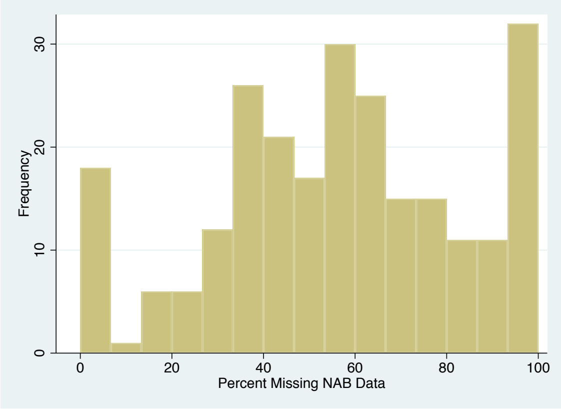

B. Gaps in NAB Data

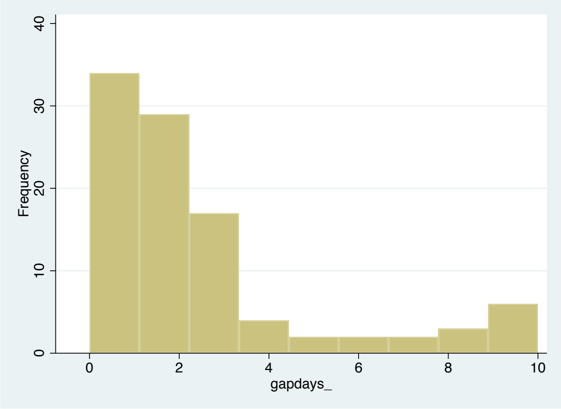

C. First Available NAB Data

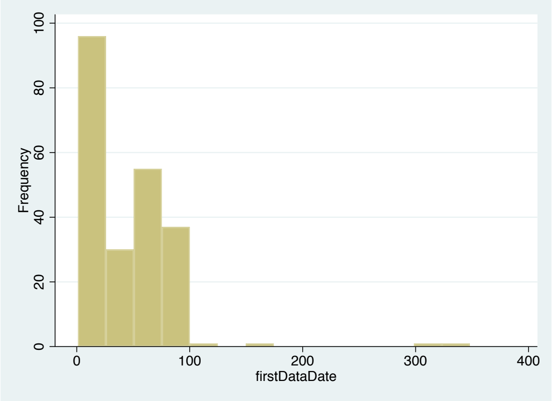

Supplementary Figure 6. Google Trends data quality. A. Range of percent days of missing GT data by station-year, B. Missing GT data over time.

A. Missing GT data for all station-years

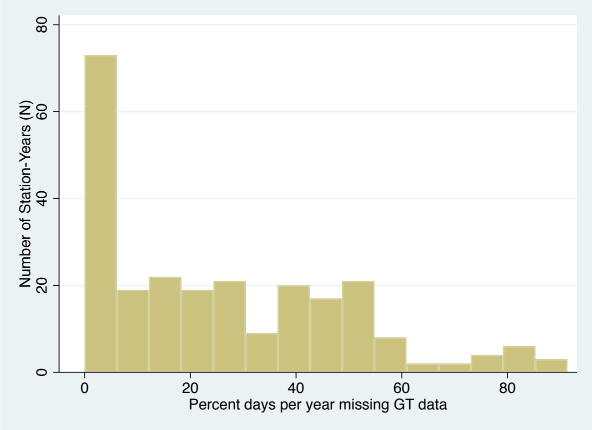

B. Median percent missing GT data over time

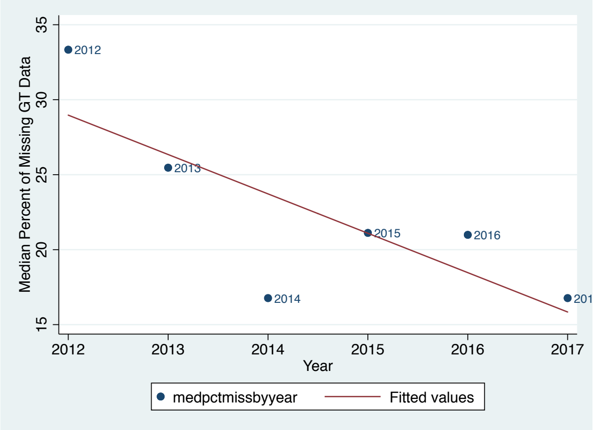

Supplementary Figure 7. By Ecoregion: NAB Data Quality, and Correlation Strength between NAB and GT data.

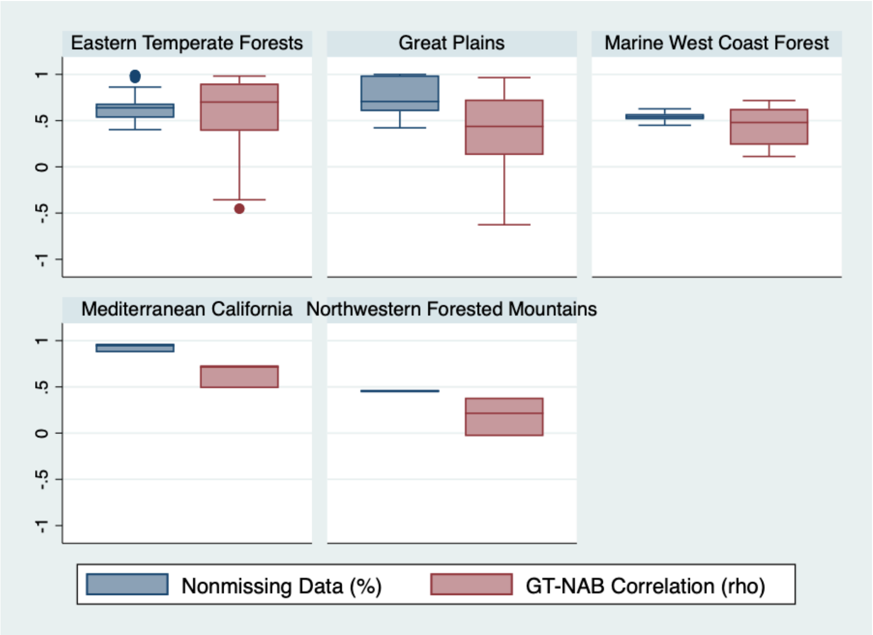

Supplementary Figure 8. Relationship between mean spring temperature and availability of daily pollen concentration data.

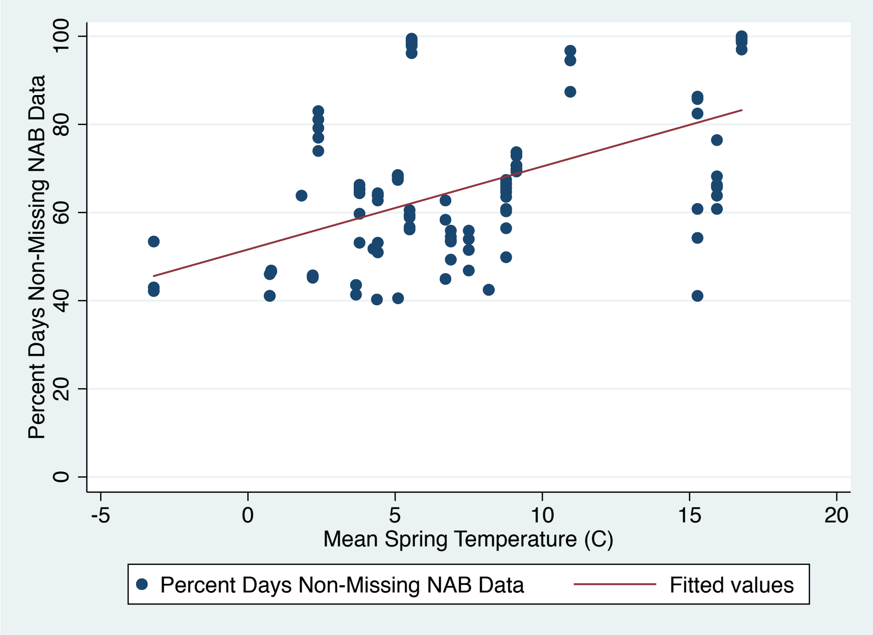

Supplementary Figure 9. Relationship between GT data quality (percent days missing) and regional media consumption (log TV-homes).

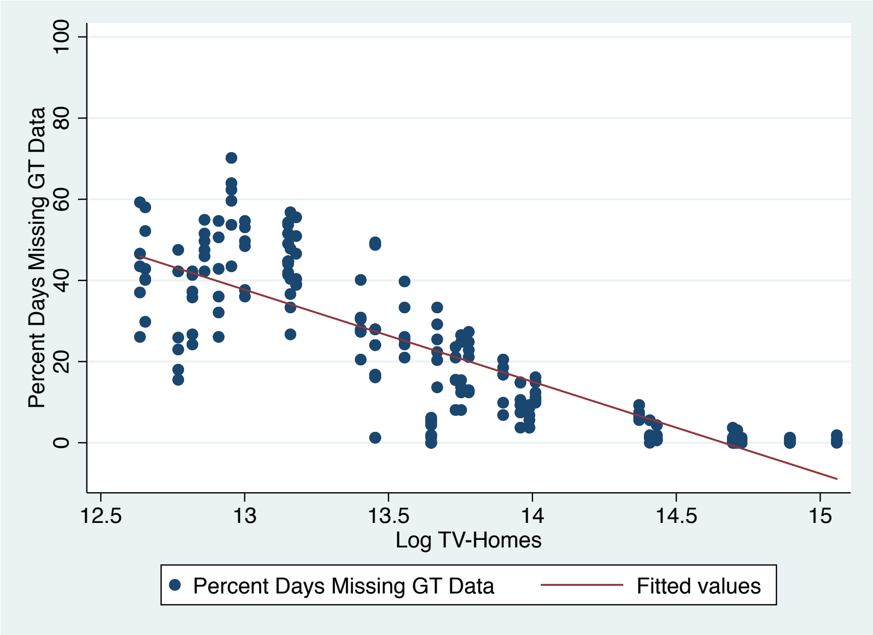

Supplementary Figure 10. Ecoregion classifications of NAB stations meeting data quality inclusion criteria for correlation analyses with GT data in the current study

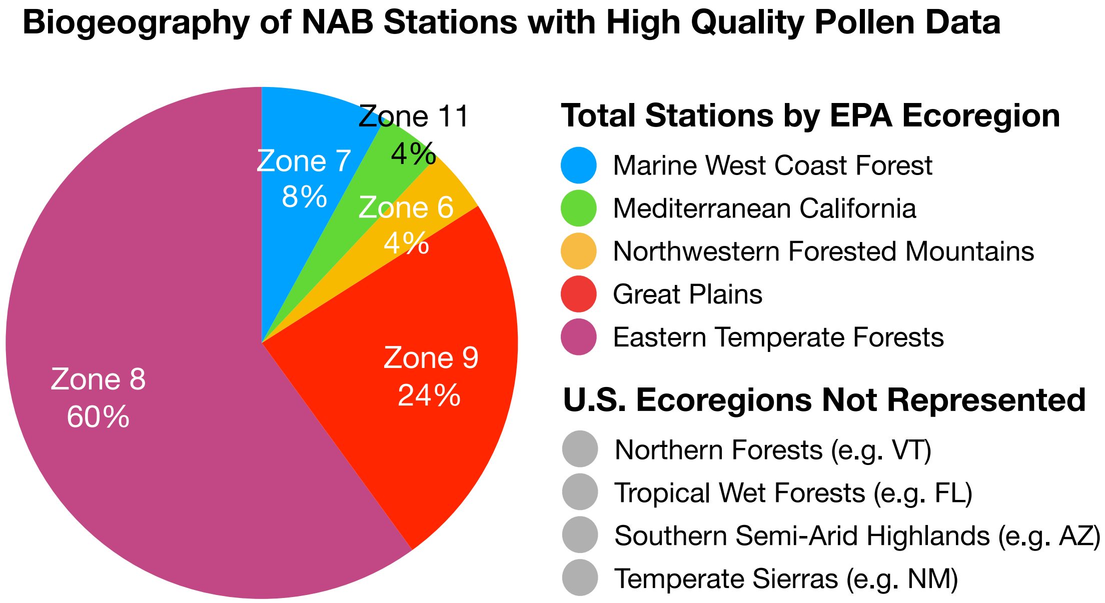

Supplementary Figure 11. Google Trends Data Coverage in NAB Observation-Free Zones. Markers show locations with available data on pollen patterns from A. NAB stations meeting data quality inclusion criteria for this study (red), B. Google Trends DMA regions ranked in the top 50 for number of TV-Homes (blue), and C. All other Google Trends DMA regions of any rank (yellow). Interactive map may be accessed at: http://bit.ly/2XTlHrC

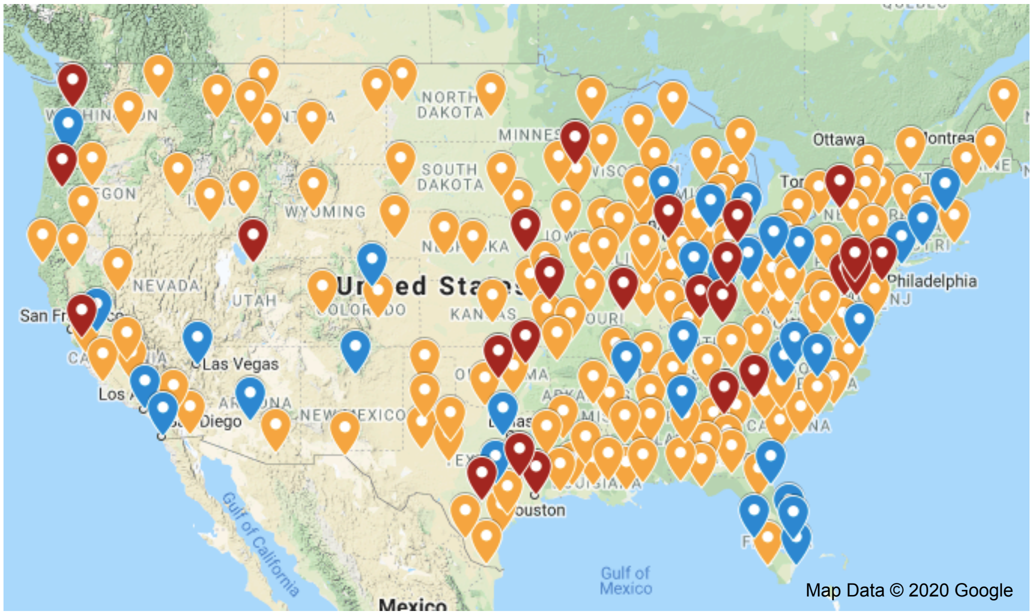

Supplementary Figure 12. Evaluating strength of peak signal in Google Trends searches. A. Representative example of strong GT peak signal relative to a heavily-smoothing lowess function (bandwidth = 0.8). B. Correlation between percent of missing GT data and peak signal strength.

A. Peak signal: deviation from lowess

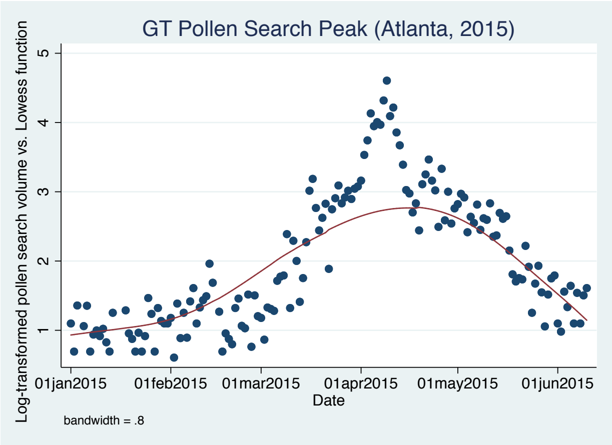

B. Peak signal vs. percent missing GT data

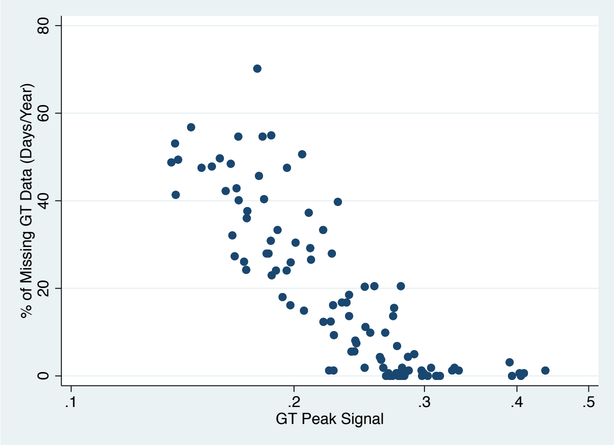

Spearman's rho: -0.87; p < 0.01

Supplementary Figure 13. A. Example year, 2013, GT-NAB correlation strength by precipitation B. GT-NAB correlation strength by spring temperature

A. Total annual precipitation (days) B. Mean spring temperature (°C)

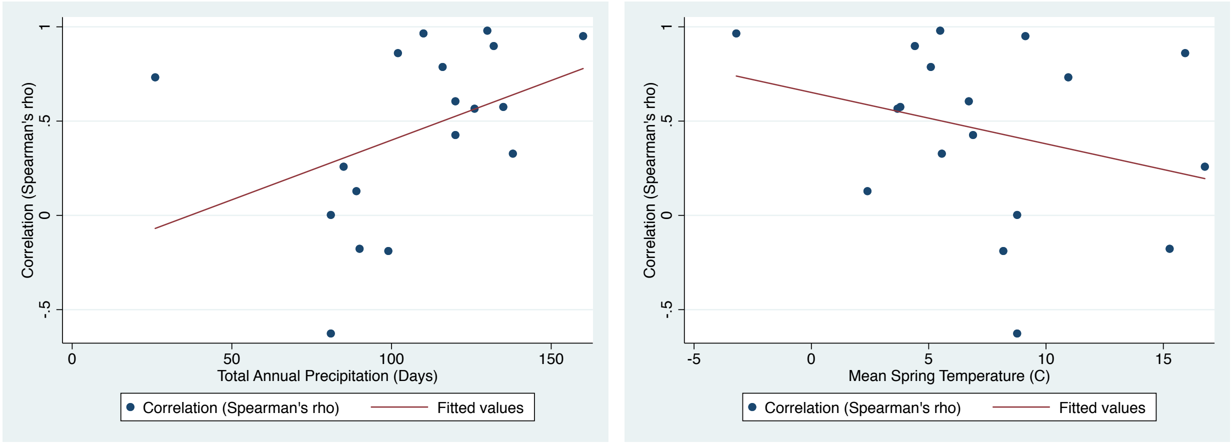

Supplementary Figure 14. DMA rank vs. GT Missingness

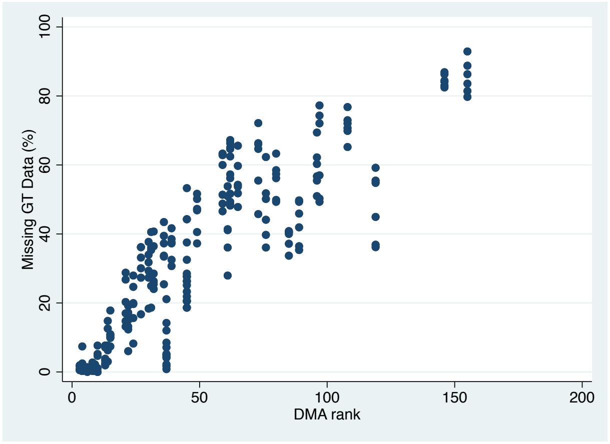

$R^2 = 0.78; p < 0.01$

Supplementary Tables

Supplementary Table 1. Complete list of candidate search terms from relevant literature evaluated in this study.

Search Term	Reference	Notes
Pollen	Bousquet et al., 2019, Willson et al., 2015	Term used in the current study
Rhinitis	Bousquet et al., 2019 Kang et al., 2015, Marques, 2016	Not distinct from "allergic rhinitis" and "hay fever" in GT; low relative search volume
Runny nose, congestion	Willson et al., 2015	Peaks in Dec-Feb, appearing to follow cold/flu seasonality
Allergy	Bousquet et al., 2019 Willson et al., 2015	Closely mirrors search pattern for "pollen"
Asthma	Bousquet et al., 2019, Marques 2016	Exhibited no seasonality
Conjunctivitis	Bousquet et al., 2019	Exhibited no seasonality
Hay fever	Bousquet et al., 2019, Kang et al., 2015	Very low search volume
Allergic conjunctivitis, ocular allergy, eye allergy, rose fever, pollen allergy	Kang et al., 2015	Very low search volume
Post nasal drainage	Willson et al., 2015	No seasonality, very low search volume

Supplementary Table 2. GT DMA-Matched NAB locations, rank-correlation coefficients, and covariates by station-year.
** Missing Rho values represent insufficient GT and NAB data for rank-correlation analyses.*

NAB Station	State	Matched DMA (Code)	Matched DMA Name	Year	Spearman's Rho	NAB % Missing	NAB Longest Gap	GT % Missing	GT Peak Signal Decile
Atlanta	GA	524	Atlanta	2012	0.96	26.3	2	1.2	10
Atlanta	GA	524	Atlanta	2013	0.95	27.1	2	0.0	10
Atlanta	GA	524	Atlanta	2014	0.97	30.1	2	0.6	10
Atlanta	GA	524	Atlanta	2015	0.98	29.3	2	3.1	10
Atlanta	GA	524	Atlanta	2016	0.98	30.7	3	0.6	10
Atlanta	GA	524	Atlanta	2017	0.90	29.3	2	0.0	10
Baltimore	MD	512	Baltimore	2012	0.80	35.6	1	18.5	6
Baltimore	MD	512	Baltimore	2013	0.90	46.8	0	20.5	9
Baltimore	MD	512	Baltimore	2014	0.80	37.3	0	16.8	6
Baltimore	MD	512	Baltimore	2015	0.83	36.2	0	20.5	8
Baltimore	MD	512	Baltimore	2016	0.43	36.2	3	9.9	8
Baltimore	MD	512	Baltimore	2017	0.79	49.0	3	6.8	9
Bellevue	NE	652	Omaha	2012	-0.31	18.9	1	50.6	5
Bellevue	NE	652	Omaha	2013	0.13	26.0	0	54.7	3
Bellevue	NE	652	Omaha	2014	0.16	17.0	1	42.9	3
Bellevue	NE	652	Omaha	2015	0.47	24.4	6	36.0	3
Bellevue	NE	652	Omaha	2016	-0.23	23.0	1	32.1	3
Bellevue	NE	652	Omaha	2017	0.47	20.8	2	26.1	3
Charleston2	SC	519	Charleston, SC	2012		100.0	0	59.3	1
Charleston2	SC	519	Charleston, SC	2013		100.0	0	46.6	1
Charleston2	SC	519	Charleston, SC	2014		100.0	0	46.6	3
Charleston2	SC	519	Charleston, SC	2015		100.0	0	43.5	5
Charleston2	SC	519	Charleston, SC	2016	0.94	62.2	2	37.0	6
Charleston2	SC	519	Charleston, SC	2017	0.69	60.0	2	26.1	6
Charlotte	NC	517	Charlotte	2012	0.90	76.7	9	14.8	9
Charlotte	NC	517	Charlotte	2013	0.48	88.8	10	7.5	10
Charlotte	NC	517	Charlotte	2014	0.81	73.7	8	10.6	8
Charlotte	NC	517	Charlotte	2015	0.82	79.2	10	14.9	9
Charlotte	NC	517	Charlotte	2016	0.95	72.3	5	9.3	9
Charlotte	NC	517	Charlotte	2017	0.52	77.0	10	3.7	9
CollegeStation	TX	625	Waco / Temple / Bryan	2012	-0.09	39.2	2	47.5	4
CollegeStation	TX	625	Waco / Temple / Bryan	2013	-0.18	17.5	2	42.2	2
CollegeStation	TX	625	Waco / Temple / Bryan	2014	0.04	13.7	1	15.5	8
CollegeStation	TX	625	Waco / Temple / Bryan	2015	-0.21	14.2	1	23.0	4
CollegeStation	TX	625	Waco / Temple / Bryan	2016	-0.36	45.8	1	25.9	5
CollegeStation	TX	625	Waco / Temple / Bryan	2017	-0.45	58.9	0	18.0	4
Dayton	OH	542	Dayton	2012	0.23	35.6	2	53.1	1
Dayton	OH	542	Dayton	2013	0.58	34.8	2	48.4	3

Dayton	OH	542	Dayton	2014	0.65	34.5	2	49.7	2
Dayton	OH	542	Dayton	2015	0.29	33.7	2	54.7	4
Dayton	OH	542	Dayton	2016	0.06	40.3	2	37.7	3
Dayton	OH	542	Dayton	2017	0.57	46.8	3	36.0	3
Draper	UT	770	Salt Lake City	2012	0.21	54.8	2	26.5	5
Draper	UT	770	Salt Lake City	2013	0.04	57.3	6	24.8	6
Draper	UT	770	Salt Lake City	2014	-0.03	54.2	2	13.7	8
Draper	UT	770	Salt Lake City	2015	0.38	54.5	2	12.4	6
Draper	UT	770	Salt Lake City	2016	0.49	57.0	5	15.4	7
Draper	UT	770	Salt Lake City	2017	0.29	60.5	2	8.1	6
Erie	PA	516	Erie	2012		68.8	8	88.9	1
Erie	PA	516	Erie	2013	-0.14	65.2	3	80.7	1
Erie	PA	516	Erie	2014		67.1	3	85.1	1
Erie	PA	516	Erie	2015	0.48	68.5	9	84.5	1
Erie	PA	516	Erie	2016	0.33	63.3	7	79.0	1
Erie	PA	516	Erie	2017	0.51	66.0	10	80.1	4
Eugene	OR	801	Eugene	2012	0.23	37.3	0	45.7	3
Eugene	OR	801	Eugene	2013	0.61	41.6	4	37.3	5
Eugene	OR	801	Eugene	2014	0.62	44.1	6	31.1	6
Eugene	OR	801	Eugene	2015	0.65	55.1	3	40.4	4
Eugene	OR	801	Eugene	2016	0.32	56.7	5	23.5	7
Eugene	OR	801	Eugene	2017		100.0	0	28.6	7
Greenville	SC	567	Greenvll / Spart / Ashevll / And	2012	0.62	53.2	4	33.3	6
Greenville	SC	567	Greenvll / Spart / Ashevll / And	2013	0.80	47.9	8	25.5	5
Greenville	SC	567	Greenvll / Spart / Ashevll / And	2014	0.49	48.5	7	22.4	5
Greenville	SC	567	Greenvll / Spart / Ashevll / And	2015	0.57	48.5	4	29.2	5
Greenville	SC	567	Greenvll / Spart / Ashevll / And	2016	0.77	46.0	2	20.4	7
Greenville	SC	567	Greenvll / Spart / Ashevll / And	2017	0.39	44.1	3	13.7	6
Houston	TX	618	Houston	2012	0.70	33.7	3	3.7	8
Houston	TX	618	Houston	2013	0.86	34.2	2	0.0	8
Houston	TX	618	Houston	2014	0.90	36.2	2	0.6	9
Houston	TX	618	Houston	2015	0.53	39.2	2	1.2	9
Houston	TX	618	Houston	2016	0.95	31.8	3	0.0	9
Houston	TX	618	Houston	2017	0.93	23.6	0	0.0	8
KansasCity	MO	616	Kansas City	2012	0.51	51.5	9	21.0	4
KansasCity	MO	616	Kansas City	2013	0.62	52.6	10	15.5	5
KansasCity	MO	616	Kansas City	2014	0.66	53.4	9	23.6	5
KansasCity	MO	616	Kansas City	2015	0.47	51.0	10	21.1	5
KansasCity	MO	616	Kansas City	2016	0.69	49.3	10	15.4	5
KansasCity	MO	616	Kansas City	2017	0.65	48.2	2	8.1	7
Knoxville	TN	557	Knoxville	2012	0.18	80.5	8	55.6	2
Knoxville	TN	557	Knoxville	2013	0.67	80.5	4	40.4	4
Knoxville	TN	557	Knoxville	2014	0.22	81.1	4	46.6	3
Knoxville	TN	557	Knoxville	2015	0.43	80.3	10	50.9	3
Knoxville	TN	557	Knoxville	2016	0.85	80.5	6	38.9	3
Knoxville	TN	557	Knoxville	2017		100.0	0	39.1	3
Lexington1	KY	541	Lexington	2012	0.48	74.8	3	54.3	2
Lexington1	KY	541	Lexington	2013	-0.05	60.5	2	51.6	2
Lexington1	KY	541	Lexington	2014	-0.18	67.4	2	49.1	1

Lexington1	KY	541	Lexington	2015	-0.34	61.9	2	44.7	2
Lexington1	KY	541	Lexington	2016	-0.01	59.5	0	41.4	2
Lexington1	KY	541	Lexington	2017	-0.42	66.6	0	44.7	2
Lexington2	KY	541	Lexington	2012		95.6	0	53.7	2
Lexington2	KY	541	Lexington	2013	0.35	67.4	4	51.6	2
Lexington2	KY	541	Lexington	2014	0.14	80.5	3	49.1	1
Lexington2	KY	541	Lexington	2015	-0.01	74.2	7	44.7	2
Lexington2	KY	541	Lexington	2016	0.01	83.0	2	42.0	1
Lexington2	KY	541	Lexington	2017	0.56	77.0	8	44.1	2
Louisville	KY	529	Louisville	2012	0.73	2.2	1	40.1	3
Louisville	KY	529	Louisville	2013	0.33	1.1	1	27.3	3
Louisville	KY	529	Louisville	2014	0.63	1.6	1	30.4	5
Louisville	KY	529	Louisville	2015	0.67	3.8	0	28.0	6
Louisville	KY	529	Louisville	2016	0.24	0.5	0	30.9	4
Louisville	KY	529	Louisville	2017		100.0	0	20.5	4
Madison	WI	669	Madison	2012	0.76	64.7	5	41.4	4
Madison	WI	669	Madison	2013	0.73	69.6	3	42.2	6
Madison	WI	669	Madison	2014	0.65	67.9	4	37.3	5
Madison	WI	669	Madison	2015	0.48	65.2	3	26.7	4
Madison	WI	669	Madison	2016	0.11	61.1	10	35.8	4
Madison	WI	669	Madison	2017	0.72	65.5	4	24.2	3
MelrosePark	IL	602	Chicago	2012	0.54	58.4	7	0.6	9
MelrosePark	IL	602	Chicago	2013	0.85	62.5	2	0.6	7
MelrosePark	IL	602	Chicago	2014	0.21	62.7	2	0.6	7
MelrosePark	IL	602	Chicago	2015	0.93	58.9	3	1.9	7
MelrosePark	IL	602	Chicago	2016	0.67	58.9	2	0.6	10
MelrosePark	IL	602	Chicago	2017	0.83	54.0	2	0.0	8
Midland	TX	633	Odessa / Midland	2012		100.0	0	91.4	1
Midland	TX	633	Odessa / Midland	2013		100.0	0	90.1	1
Midland	TX	633	Odessa / Midland	2014	-0.50	86.3	10	82.6	1
Midland	TX	633	Odessa / Midland	2015	-0.31	74.8	4	82.6	1
Midland	TX	633	Odessa / Midland	2016	-0.39	75.6	6	75.3	1
Midland	TX	633	Odessa / Midland	2017	0.18	79.5	4	72.0	1
Minneapolis	MN	613	Minneapolis / St. Paul	2012	0.68	42.2	9	9.3	7
Minneapolis	MN	613	Minneapolis / St. Paul	2013	0.96	46.6	2	9.3	6
Minneapolis	MN	613	Minneapolis / St. Paul	2014	0.79	57.0	2	7.5	7
Minneapolis	MN	613	Minneapolis / St. Paul	2015	0.69	57.8	10	6.8	7
Minneapolis	MN	613	Minneapolis / St. Paul	2016	0.69	40.3	10	6.2	8
Minneapolis	MN	613	Minneapolis / St. Paul	2017	0.60	57.8	3	5.6	7
MountLaurel	NJ	504	Philadelphia	2012	0.78	71.5	5	0.6	7
MountLaurel	NJ	504	Philadelphia	2013	0.87	73.4	3	0.0	6

MountLaurel	NJ	504	Philadelphia	2014	0.82	73.2	10	0.6	7
MountLaurel	NJ	504	Philadelphia	2015	0.90	60.3	3	1.2	9
MountLaurel	NJ	504	Philadelphia	2016	0.81	57.5	8	0.0	7
MountLaurel	NJ	504	Philadelphia	2017		100.0	0	0.6	10
OklahomaCity1	OK	650	Oklahoma City	2012	0.16	34.5	2	48.8	1
OklahomaCity1	OK	650	Oklahoma City	2013	0.00	50.1	3	28.0	4
OklahomaCity1	OK	650	Oklahoma City	2014	0.17	37.8	8	16.1	5
OklahomaCity1	OK	650	Oklahoma City	2015	0.73	36.4	2	1.2	6
OklahomaCity1	OK	650	Oklahoma City	2016	0.28	33.4	2	24.1	4
OklahomaCity1	OK	650	Oklahoma City	2017	0.41	33.7	2	16.8	6
OklahomaCity2	OK	650	Oklahoma City	2012	0.26	32.6	2	49.4	2
OklahomaCity2	OK	650	Oklahoma City	2013	-0.63	39.2	3	28.0	4
OklahomaCity2	OK	650	Oklahoma City	2014	0.19	35.3	2	16.1	5
OklahomaCity2	OK	650	Oklahoma City	2015	0.73	43.6	3	1.2	6
OklahomaCity2	OK	650	Oklahoma City	2016	0.47	39.7	2	24.1	4
OklahomaCity2	OK	650	Oklahoma City	2017	0.48	67.4	3	16.8	6
OklahomaCity3	OK	650	Oklahoma City	2012		100.0	0	48.8	2
OklahomaCity3	OK	650	Oklahoma City	2013		100.0	0	28.0	4
OklahomaCity3	OK	650	Oklahoma City	2014		100.0	0	16.1	5
OklahomaCity3	OK	650	Oklahoma City	2015		97.5	0	1.2	6
OklahomaCity3	OK	650	Oklahoma City	2016	0.37	54.2	8	24.1	4
OklahomaCity3	OK	650	Oklahoma City	2017	0.49	73.4	4	16.8	6
Philadelphia	PA	504	Philadelphia	2012	0.81	74.2	10	0.6	7
Philadelphia	PA	504	Philadelphia	2013	0.80	74.0	3	0.0	6
Philadelphia	PA	504	Philadelphia	2014	0.92	73.4	3	0.6	7
Philadelphia	PA	504	Philadelphia	2015	0.90	59.7	2	1.2	9
Philadelphia	PA	504	Philadelphia	2016	0.76	56.4	7	0.0	8
Philadelphia	PA	504	Philadelphia	2017	0.93	53.2	5	0.6	10
Rochester	NY	538	Rochester, NY	2012	-0.35	53.2	3	54.9	4
Rochester	NY	538	Rochester, NY	2013	0.43	63.3	2	49.7	4
Rochester	NY	538	Rochester, NY	2014	-0.25	64.1	4	46.0	2
Rochester	NY	538	Rochester, NY	2015	0.00	63.0	2	51.6	3
Rochester	NY	538	Rochester, NY	2016	0.71	53.4	4	47.5	2
Rochester	NY	538	Rochester, NY	2017	0.45	53.4	8	42.2	1

SaintLouis	MO	609	St. Louis	2012	0.77	31.8	3	12.3	6
SaintLouis	MO	609	St. Louis	2013	0.79	31.5	2	16.1	6
SaintLouis	MO	609	St. Louis	2014	0.94	32.6	2	11.2	7
SaintLouis	MO	609	St. Louis	2015	0.91	32.3	3	14.9	5
SaintLouis	MO	609	St. Louis	2016	0.66	31.5	3	9.9	7
SaintLouis	MO	609	St. Louis	2017		100.0	0	10.6	8
SanAntonio 2	TX	641	San Antonio	2012	0.04	0.0	0	5.6	7
SanAntonio 2	TX	641	San Antonio	2013	0.26	0.5	0	1.9	9
SanAntonio 2	TX	641	San Antonio	2014	0.55	1.4	3	1.9	10
SanAntonio 2	TX	641	San Antonio	2015	0.76	0.3	0	5.0	9
SanAntonio 2	TX	641	San Antonio	2016	0.57	0.3	0	0.0	10
SanAntonio 2	TX	641	San Antonio	2017	0.69	3.0	0	0.0	9
SanAntonio 3	TX	641	San Antonio	2012		100.0	0	6.2	7
SanAntonio 3	TX	641	San Antonio	2013		100.0	0	1.9	9
SanAntonio 3	TX	641	San Antonio	2014	0.84	0.8	0	1.2	10
SanAntonio 3	TX	641	San Antonio	2015	0.85	0.5	0	4.3	9
SanAntonio 3	TX	641	San Antonio	2016	0.77	1.1	0	0.0	10
SanAntonio 3	TX	641	San Antonio	2017	0.77	1.1	0	0.0	9
SanJose	CA	807	San Francisco / Oak / San Jose	2012	0.49	3.3	0	0.6	8
SanJose	CA	807	San Francisco / Oak / San Jose	2013	0.73	12.6	0	0.0	9
SanJose	CA	807	San Francisco / Oak / San Jose	2014	-0.23	41.4	8	0.0	7
SanJose	CA	807	San Francisco / Oak / San Jose	2015	0.30	6.6	5	1.2	8
SanJose	CA	807	San Francisco / Oak / San Jose	2016	0.71	5.5	0	0.0	9
SanJose	CA	807	San Francisco / Oak / San Jose	2017	0.61	19.2	7	0.0	8
Savannah	GA	507	Savannah	2012		92.6	10	58.0	2
Savannah	GA	507	Savannah	2013	-0.43	87.9	7	52.2	2
Savannah	GA	507	Savannah	2014		98.6	10	40.4	4
Savannah	GA	507	Savannah	2015		96.7	9	42.9	3
Savannah	GA	507	Savannah	2016	0.48	91.8	10	40.1	5
Savannah	GA	507	Savannah	2017		91.2	10	29.8	4
Seattle	WA	819	Seattle / Tacoma	2012	0.11	46.3	3	4.3	8
Seattle	WA	819	Seattle / Tacoma	2013	0.43	46.6	0	1.2	10
Seattle	WA	819	Seattle / Tacoma	2014	0.19	41.4	10	0.6	9
Seattle	WA	819	Seattle / Tacoma	2015	0.25	50.7	2	1.9	8

Seattle	WA	819	Seattle / Tacoma	2016	0.53	44.1	0	0.6	9
Seattle	WA	819	Seattle / Tacoma	2017	0.72	45.5	0	0.6	9
Sparks	NV	811	Reno	2012	0.21	89.3	10	75.9	1
Sparks	NV	811	Reno	2013	0.49	86.3	7	75.8	1
Sparks	NV	811	Reno	2014		95.3	10	52.8	2
Sparks	NV	811	Reno	2015		92.9	6	54.0	1
Sparks	NV	811	Reno	2016	-0.28	84.7	6	56.8	1
Sparks	NV	811	Reno	2017	-0.24	83.3	10	55.9	1
Sylvania	OH	547	Toledo	2012		100.0	0	62.3	2
Sylvania	OH	547	Toledo	2013		100.0	0	64.0	2
Sylvania	OH	547	Toledo	2014	0.66	38.6	5	59.6	3
Sylvania	OH	547	Toledo	2015	0.59	36.2	0	70.2	3
Sylvania	OH	547	Toledo	2016	0.58	43.8	9	53.7	1
Sylvania	OH	547	Toledo	2017	0.33	39.5	9	43.5	3
Tampa	FL	539	Tampa / St. Pete (Sarasota)	2012		96.4	10	5.6	7
Tampa	FL	539	Tampa / St. Pete (Sarasota)	2013		100.0	0	1.9	8
Tampa	FL	539	Tampa / St. Pete (Sarasota)	2014		100.0	0	1.2	8
Tampa	FL	539	Tampa / St. Pete (Sarasota)	2015		100.0	0	1.2	10
Tampa	FL	539	Tampa / St. Pete (Sarasota)	2016	0.75	88.2	9	1.2	9
Tampa	FL	539	Tampa / St. Pete (Sarasota)	2017	0.70	88.5	6	0.0	8
Tulsa	OK	671	Tulsa	2012	0.09	57.5	4	56.8	2
Tulsa	OK	671	Tulsa	2013	-0.19	57.5	3	47.8	2
Tulsa	OK	671	Tulsa	2014	0.20	52.9	7	36.6	2
Tulsa	OK	671	Tulsa	2015	0.65	68.2	6	40.4	5
Tulsa	OK	671	Tulsa	2016	0.02	57.5	10	33.3	2
Tulsa	OK	671	Tulsa	2017	0.18	55.3	8	26.7	3
TwinFalls	ID	760	Twin Falls						
Vancouver	WA	820	Portland, OR	2012		100.0	0	8.6	6
Vancouver	WA	820	Portland, OR	2013		100.0	0	9.3	9
Vancouver	WA	820	Portland, OR	2014		100.0	0	6.8	10
Vancouver	WA	820	Portland, OR	2015	0.07	88.5	6	3.7	7
Vancouver	WA	820	Portland, OR	2016	0.48	88.8	10	5.6	8
Vancouver	WA	820	Portland, OR	2017		96.4	0	3.7	8
Washington	MD	511	Washington, DC (Hagrstwn)	2012	0.95	41.1	4	1.9	10
Washington	MD	511	Washington, DC (Hagrstwn)	2013	0.98	43.3	3	0.0	10
Washington	MD	511	Washington, DC (Hagrstwn)	2014	0.98	43.8	4	0.0	10
Washington	MD	511	Washington, DC (Hagrstwn)	2015	0.83	39.5	2	0.0	10
Washington	MD	511	Washington, DC (Hagrstwn)	2016	0.89	40.5	3	1.2	10

Washington	MD	511	Washington, DC (Hagrstwn)	2017		100.0	0	0.0	10
Waterbury	CT	533	Hartford & New Haven	2012	0.36	66.8	3	22.8	7
Waterbury	CT	533	Hartford & New Haven	2013	0.56	65.2	2	24.8	5
Waterbury	CT	533	Hartford & New Haven	2014	0.67	65.5	2	21.1	10
Waterbury	CT	533	Hartford & New Haven	2015	0.66	67.1	5	27.3	8
Waterbury	CT	533	Hartford & New Haven	2016	0.61	66.3	2	13.0	5
Waterbury	CT	533	Hartford & New Haven	2017	0.80	67.9	9	12.4	8
York	PA	566	Harrisburg / Lncstr / Leb / York	2012	0.16	56.4	3	33.3	4
York	PA	566	Harrisburg / Lncstr / Leb / York	2013	0.57	56.4	2	24.2	3
York	PA	566	Harrisburg / Lncstr / Leb / York	2014	0.80	63.3	3	25.5	4
York	PA	566	Harrisburg / Lncstr / Leb / York	2015	0.33	58.6	2	39.8	6
York	PA	566	Harrisburg / Lncstr / Leb / York	2016	0.71	61.1	8	21.0	5
York	PA	566	Harrisburg / Lncstr / Leb / York	2017	0.41	64.4	3	26.1	5

Supplementary Table 3. Difference between GT and NAB-derived season start dates vs. NAB historical data (days)

	N	Min	Q1	Mdn	Q3	Max
**All station-years**						
Untransformed data	105	-93	-42	-27	-14	8
Smoothed data	105	-90	-40	-26	-14	7
Smoothed log-transformed data	105	-75	-39	-24	-7	9
Previous year NAB data	84	-43	-8	2	10	40
**Inclusion Criteria: NAB data available within first month of year**						
Untransformed data	75	-68	-37	-23	-8	8
Smoothed data	75	-63	-34	-23	-7	7
Smoothed log-transformed data	75	-53	-28	-12	-3	9
Previous year NAB data	59	-38	-8	2	12	37
**Additional inclusion criteria: Less than 20% GT data missing per year**						
Untransformed data	38	-62	-30	-18	-3	7
Smoothed data	38	-60	-31	-17	-2	7
Smoothed log-transformed data	38	-52	-21	-8.5	0	5
Previous year NAB data	31	-23	-7	2	8	24
